# Designing a mobile health smokeless tobacco cessation intervention in Odisha, India: User and provider perspectives

**DOI:** 10.1177/20552076221150581

**Published:** 2023-01-11

**Authors:** Rajmohan Panda, Supriya Lahoti, Arti Mishra, Rajath R Prabhu, Sangeeta Das, Durga Madhab Satapathy, Irwin Nazareth

**Affiliations:** 1 Research Division, 119663 Public Health Foundation of India, New Delhi, India; 2 Project ECHO (Extension for Community Healthcare Outcomes), New Delhi, India; 3 Research Division, Public Health Foundation of India, India; 4 Department of Primary Care and Population Sciences, University College London, London, UK

**Keywords:** Qualitative research, mobile health, smokeless tobacco, intervention, primary care, India

## Abstract

**Objective:**

There is limited evidence on the development of mobile health (mHealth) interventions for smokeless tobacco (SLT) cessation, despite its widespread use in South Asia. This formative qualitative study explored the perceptions of tobacco users and healthcare providers (HCPs) regarding developing a mHealth intervention for SLT cessation.

**Methods:**

This was a qualitative study using in-depth interviews (IDIs) with tobacco users (n = 26) and primary care physicians (PCPs) (n = 5) and focus group discussions (FGDs) with counsellors (n = 2) in four urban primary health centres (UPHCs) in Berhampur, Odisha from February to March 2020. The data were coded and analysed by two researchers using a framework analysis method. The discussion guides and initial codes were developed based on the Transtheoretical Model (TTM) of behaviour change.

**Results:**

The results were elaborated under four themes: (1) Current scenario of SLT use; (2) Barriers and facilitators for quitting SLT; (3) Barriers and facilitators for mHealth counselling; and (4) Design and delivery of the proposed intervention. SLT use was prevalent in the community regardless of sociodemographic factors. Peer factors accounted for both tobacco consumption as well as considering cessation. Participants considered mobile message counselling helpful and acceptable. Not having a mobile phone and illiteracy were identified as barriers while ease of access and rising popularity of social media applications were considered facilitators to the use of mHealth for quitting tobacco. Participants preferred messages that were pictorial, short and simple, in the local language, and tailored to individual's needs.

**Conclusions:**

This is the first study that provides evidence within the Indian context that the text messaging platform may be used for delivering an SLT cessation intervention. The integration of a theoretical basis and research findings from target users can guide future intervention development.

## Introduction

Tobacco use is globally responsible for almost eight million deaths each year or a death every 6 seconds.^1, 2^ In India, the tobacco problem is complex and most tobacco users use a variety of tobacco products – combustible, non-combustible or both. India and Bangladesh alone account for 232 of 248 million worldwide smokeless tobacco (SLT) users.^3^ According to the Indian Global Adult Tobacco Survey (GATS 2016–2017), 199.4 million adults (21.4% overall, 29.6% men and 12.8% women) consume SLT regularly.^4^ The all-cause mortality attributable to SLT use in India in 2009–2010 was estimated to be 368,127 people, with nearly three-fifths of these deaths occurring in women (i.e. 217,076 women and 151,051 men).^5^ In India, the highest prevalence of SLT use has been observed in the north-eastern and eastern states of Tripura (48.5%) and Odisha (42.9%).^4^

Legislation such as the Cigarettes and Other Tobacco Products Act (COTPA 2003)^6^ have been introduced in India to curb the use of SLT but these are poorly implemented.^7,8^ One intervention for SLT cessation could be the use of mobile messaging with its wide reach, ability to overcome geographic barriers,^9^ low cost and potential for scalability.^10^ India has high rates of mobile phone ownership with 1153.7 million subscribers in 2020 (629.7 million urban and 524 million rural subscribers),^11^ making mobile health (mHealth) interventions an attractive option to promote cessation. Mobile messaging allows users to access short-messaging services (SMS) whenever they feel an urge to use tobacco and these can be delivered to users in real time. These messages can be tailored to individual needs while also interacting with the end user hence changing behaviour and achieving effective tobacco cessation.^7,10^

Behaviour change models such as the Transtheoretical Model (TTM) by Prochaska and Velicer have been used to develop tobacco cessation interventions.^12,13^ This model operates on the assumption that people do not change habitual behaviours such as SLT consumption quickly and decisively and people will quit SLT when they are ready to do so.^14^ Behavioural interventions have overall efficacy in SLT cessation in adults (Risk ratio = 1.63, 95% confidence interval = 1.32 to 1.94) both in high and low-income countries.^15^ The acceptability, appeal and effectiveness of these interventions can be maximized by engaging the user in its design and development.^16^ In our study, we sought the views of healthcare providers (HCPs) and tobacco users in primary care to inform the design of the intervention of the Counselling intErvention foR smokeless Tobacco cessAtion in INdian primary care (CERTAIN) trial. The details of the randomized trial protocol are provided in a separate paper.^17^ The specific objectives of this formative research were to explore perceptions of HCPs and tobacco users’ on:

- Current scenario of SLT use;
- Barriers and facilitators faced by tobacco users in quitting SLT;
- Barriers and facilitators for participation in mHealth counselling for SLT cessation; and
- Design and delivery of the proposed intervention for SLT cessation.

## Methods

### Study design and setting

A qualitative study was conducted to address these research questions. This included in-depth interviews (IDIs) and focus group discussions (FGDs), to get a more comprehensive evaluation than could be gained by a single method of inquiry. The study was conducted in four urban primary health centres (UPHCs) situated in Berhampur city in Odisha, India. We included UPHCs serving varied populations such as semi-urban, women, low-income and elderly as well as mixed populations.

### Sampling of participants

We employed a combination of purposive criterion-based and maximum variation sampling strategies to capture perceptions from different HCPs and tobacco users across gender, age and type of tobacco use. This ensured that the sample included a diverse group of people in terms of professional experience, program implementation and types of tobacco users. There were two categories of respondents, the HCPs working in the UPHCs, that is, primary care physician (PCP) (n = 5) and counsellor (n = 12) and the patient using tobacco (n = 26). A total of 31 IDIs and two FGDs were conducted. IDIs were conducted with PCPs and tobacco users while FGDs were conducted with counsellors. Each focus group had six participants. The inclusion criteria for HCPs were that they were employed at the UPHC for more than a year and were willing to be interviewed. The tobacco users were aged 18 years or over and were either SLT users or dual users (i.e. smokers and SLT users). Tobacco users were recruited through key informants {PCPs, clerks, Accredited Social Health Activists (ASHAs) and auxiliary nurse midwives (ANMs)^18^ posted in the UPHCs} and snowball sampling, that is, an SLT user suggested other known users in the UPHC. The eligible users were approached in person and were provided a participant information sheet (PIS) that included details of the study. Participants provided written consent before the study.

### Data collection

Data were collected from February to March 2020 by a team of field investigators trained in qualitative data collection. All investigators (resident doctors with an MD degree) were trained by the authors and had been involved in conducting qualitative research studies. The interviewers were keen on building skills in formative research. Some of the IDIs and FGDs were conducted by the authors (RP and SD). Separate discussion guides (that included a list of topics, open-ended questions and prompts), were developed for the IDIs (Appendices 1 and 2) and the FGDs, respectively (Appendix 3). These guides were developed based on a review of the literature, consultation with experts and TTM of behaviour change. The guides were developed in English, translated into Odia, and piloted with SLT users as well as doctors and counsellors. Both IDIs and FGDs were conducted in the UPHC and lasted 30–45 minutes. Field notes were prepared by the investigators during the conduct of the interviews and focus groups. We stopped collecting data at the point of data saturation when no new information was generated. All IDIs and FGDs were audio recorded using a digital recorder, conducted in the local language Odia and translated and transcribed verbatim for analysis.

### Data analysis

The findings from PCPs, counsellors and patients using tobacco were triangulated. This enabled an enhanced understanding of the tobacco problem, current interventions being offered and considerations for designing a health intervention for SLT. Triangulation was also done in a discussion between field investigators and the research team.

A framework analysis method (a matrix-based approach)^19^ was used to identify existing and new patterns in the data. This offered a systematic structure to analyse, identify and manage themes. As a first step, two authors (SL and AM) familiarized themselves with five randomly selected transcripts, and independently coded them using initial codes that were developed based on the TTM and discussion guides. We used both deductive and inductive coding, enabling potentially important themes or concepts that have been identified a priori to be combined with newly emerging themes. For instance, some of the a priori codes included age of initiation of SLT, readiness of the user to quit, previous attempts to quit, and reasons for the failure of the previous attempts. The initial double-coded transcripts were discussed and inter-coder reliability of 90% was achieved. The lead author helped develop consensus in case of discrepancies. A final codebook was developed and the coding system was applied to all the IDIs and FGDs transcripts. All transcripts were coded by two authors (SL and AM). Data saturation was achieved when no new themes emerged from the analysis of the transcripts and field notes. The themes and subthemes identified including quotes (respondents’ exact words) are presented to represent the main findings. Atlas.ti (version 8) software was used for data analysis. All ethical approvals were received from the appropriate institutional review boards.

## Results

From a total of 57 tobacco users approached, 26 agreed to participate; of these, 19 (73%) were male and 7 (27%) were female. All health professionals who were approached agreed to participate in the study. Most of the tobacco users were within the age groups between 30 and70 years. The PCPs were from between 40 and 60 years of age. Most counsellors participating in the FGDs were female (67%) from 20 to 50 years of age (Appendix 4).

The findings are broadly categorized into four different themes as follows: (1) Current scenario of SLT use; (2) Barriers and facilitators in quitting SLT; (3) Barriers and facilitators for participation in mHealth counselling; and (4) Design and delivery of the proposed intervention (Table 1).

**Table 1.** Key themes and findings.

Theme and subtheme	Key findings
Current scenario of SLT use	
Social context of SLT use	SLT use was common in the community regardless of sociodemographic factors
Knowledge of ill effects of SLT	Users were aware of SLT associated with mouth diseases.
Current status and efforts for SLT cessation	Focus is on smoking cessation Lack of training for SLT cessation Anti-tobacco advertisements increase awareness of SLT harm
Barriers and facilitators in quitting SLT as per the TTM	
Dependency on SLT (pre-contemplation stage):	Barriers: Addicted to SLT use Linked with daily routine
Challenges and support in quitting SLT (contemplation stage):	Barriers: Peer pressure for use
	Facilitators: Family and peer support Counselling by HCPs Self-motivation to quit SLT use
Challenges after attempting to quit SLT (preparation and maintenance stage):	Heavy workload Peer pressure Disturbance in daily routine
Barriers and facilitators for participation in mHealth counselling	
Perceived utility of using mobile phone counselling for SLT cessation	mHealth counselling is a good method in terms of following up SLT users
Barriers	Illiterate population and those who do not own a mobile phone
Facilitators	Trustworthy and credible when done through HCPs Use of social media designed for infotainment. Good mobile penetration in community
Design and delivery of the proposed intervention	
Content of the messages	User-friendly and context-specific Multifaceted ill effects of consumption Benefits of quitting
Design of the messages	Using social media applications Image or pictorial messages Local language Short and simple messages Not very frequent

HCP: healthcare provider; mHealth: mobile health; SLT: smokeless tobacco; TTM: Transtheoretical Model.

### Theme: Understanding the current scenario of SLT use

#### Subtheme: Social context of SLT use

The study findings revealed that the burden of SLT is high in the community, regardless of the age, economic condition and educational status of the people.

*“Tobacco (SLT) use is popular among all…below poverty line, above poverty line*^20^*” (Physician, IDI)*

*“All are having gutkha [scented tobacco mixed with lime and areca nut, in powder form] sir. Even educated people”. (Counsellor, FGD)*

*“They (smokeless tobacco users) are eating gutkha in their home, children from birth are watching that and borrow that for eating” (Counsellor, FGD)*

#### Subtheme: Knowledge of ill effects

Overall, most of the users (73%) had knowledge of SLT causing cancer and mouth diseases. However, they had inadequate and mainly hearsay knowledge. Users had little or no knowledge of the effects of SLT on chronic diseases.

*“People are telling cancer, mouth disease, these things they are telling” (Female tobacco user, IDI)*

*“It causes Cancer. Different people say different things” (Female tobacco user, IDI)*

*“Decreased B.P..Is it due to eating tobacco or what?” (Male tobacco user, IDI)*

Some of the users were unaware of the ill effects of SLT and used it regularly at home.

*“I never thought regarding diseases. Just taking gudakhu* [tobacco paste made with molasses] *twice during defecation” (Male tobacco user, IDI)*

#### Subtheme: Current status and efforts for SLT cessation

HCPs mentioned Information Education and Communication (IEC) materials and/or brief advice as tobacco cessation methods that they offer to motivate users to quit SLT.

*“We show them the posters to make them aware about SLT use, what problem one will have if he chews tobacco” (Counsellor, FGD)*

*“We are giving counselling for so many things, including tobacco cessation" (Physician, IDI)*

The views of tobacco users were sometimes discordant with the HCPs and most users mentioned that they had not received any specific intervention from HCPs to quit SLT.

*“I can't say on this matter. Whenever I have come, I haven't noticed anything like this (intervention)” (Male tobacco user, IDI)*

*“Yes, it was okay…it was casual…The doctor asked me do you chew tobacco…why don't you quit” (Male tobacco user, IDI)*

Physicians and counsellors mentioned that the current focus is more on advice against tobacco smoking; SLT cessation has largely been ignored and there is no training for counselling for SLT.

*“We do not usually address the issue of SLT during our OPD hours, only the smoking part is being focused…No training from government” (Physician, IDI)*

### Theme: Barriers and facilitators in quitting SLT

This study identified users in different stages of the TTM as well as stage-specific barriers and facilitators that users experience while quitting SLT.

#### Subtheme: Dependency on SLT (pre-contemplation stage)

Tobacco users said that they have become dependent on SLT. According to them, it has become a habit and has been linked to their daily routine work.

*“It won’t be possible that way. I am using it since childhood. The mouth will not feel good” (Female tobacco user, IDI)*

*"I am unable to quit, I mean during latrine, for smooth bowel movements" (Male tobacco user, IDI)*

#### Subtheme: Challenges and support in quitting SLT (contemplation stage)

Factors such as support from family members and friends and counselling provided by physicians can motivate the users to quit.

*“Yes, my family members have asked me to quit…my grandson who goes to English (good) medium school has urged me to quit tobacco” (Male tobacco user, IDI)*

*“Doctor along with friends will help me best to quit tobacco” (Male tobacco user, IDI)*

*“I quit alcohol as the doctor told me. Similarly, if you will ask to quit Nasha (SLT intoxication), I’ll quit that also, never take it” (Male tobacco user, IDI)*

Participants also reported peer pressure as a reason for initiation as well as the continuation of consumption of SLT.

*“My friends were giving me deliberately, they were not listening (to) me” (Male tobacco user, IDI)*

#### Subtheme: Challenges after attempting to quit SLT (preparation and maintenance stage)

The study findings indicate various reasons for relapse after the tobacco user's attempt to quit. The observed reasons for relapse were: stopping SLT consumption abruptly, workload and peer pressure.

*“As we do strenuous work, while at work my peers take tobacco in front me while I was quitting and this forced me to take tobacco again” (Male tobacco user, IDI)*

*“They have told me to leave it (SLT) slowly but I left it suddenly. I couldn’t leave it and started it again” (Male tobacco user, IDI)*

### Theme: Barriers and facilitators for participation in mHealth counselling

#### Subtheme: Perceived utility of using mobile phone counselling for SLT cessation

Respondents were of the opinion that mobile phone counselling is a good method for the delivery of cessation advice and support to SLT users.

*“If it could be in mobile, then they (family members) will tell me, you will also talk to me a little bit. Yet. It will be helpful for us as well as helpful for you.” (Female tobacco user, IDI)*

#### Subtheme: Barriers for use of mHealth counselling for SLT cessation

Few respondents identified issues that may pose challenges for mobile counselling for SLT cessation. Physicians and tobacco users mentioned that mobile counselling is good but it may be limited only to mobile phone users.

*“We cannot reach each and every person through this method” (Physician, IDI)*

An illiterate female tobacco user said she cannot read the messages.

*“I will not be able to read and understand messages; I will have to show it to my children to understand” (Female tobacco user, IDI)*

#### Subtheme: Facilitators for use of mHealth counselling for SLT cessation

Participants considered the mHealth counselling a trustworthy source of information if it comes from HCPs. They also considered it to be more accessible than face-to-face counselling.

*“Yes, yes that (mobile phone messages) will help. You will tell for our wellness” (Male tobacco user, IDI)*

*“Why to come home, it will be easier to call over the phone” (Female tobacco user, IDI)*

### Theme: Design considerations of messages for SLT cessation

#### Subtheme: Content of the messages

*Contextually tailored*: As per the physicians, messages should be context-specific and should be customized according to the tobacco user.

*“All the messages may not have same effectiveness on all people, all areas or places, message should be contextualized based on the population” (Physician, IDI)*

*Multicomponent content*: Users suggested that intervention components should emphasize ill effects of consumption, financial expenditure and benefits of quitting SLT.

*“Regarding health…what is happening by using this (SLT)…what are the benefits of quitting it” (Male tobacco user, IDI)*

*“Health benefits and various methods to help quit tobacco will be fine” (Male tobacco user, IDI)*

*“It should give information regarding extra expenditure along with ill-effects” (Male tobacco user, IDI)*

One user recommended that the content of the messages should be according to the stage of consumption or illness of those consuming SLT.

*“Cancers are caused by eating gutkha…some are in last stage, or middle or primary stage… those are in last stage should live on medicine…but those are in middle and primary stage, what should be done to quit in them and how they will be cured…” (Male tobacco user, IDI)*

*Nature of the messages*: The views of counsellors and tobacco users were in agreement regarding the content that could motivate users to think about their responsibilities towards their family members or loved ones.

*“Message should have some emotions, like if they will get the disease, there will be financial burden on them” (Counsellor, FGD)*

*“Everyone has to be made to understand that if they love their life and family members then they have to quit tobacco to have a long life.” (Male tobacco user, IDI)*

However, counsellors and SLT users had some differences regarding the inclusion of fear in messages. A counsellor stated that message content should not include words that can create fear or anger. Mobile messages should be simple, generalized to adolescents and adults and reflect the benefits of quitting.

*“We will not show any kind of anger on then. We will make them understand " (Counsellor, FGD)*

*“Almost young children from 14yrs to 30yrs are largely addicted to this…It should reflect the benefits if they quit" (Counsellor, FGD)*

Some of the users suggested that fear should be used in the messages.

*“With SLT cancer happens likewise, if we will see such messages that will make us scared to use SLT” (Male tobacco user, IDI)*

*“Message should depict harmful effects of tobacco and illnesses caused due to that, little bit scary too” (Male tobacco user, IDI)*

#### Subtheme: Design of the messages

*Use of multiple platforms*: As per the counsellors, various entertaining social media apps such as Facebook, WhatsApp and TikTok videos can be used for sharing information and making people aware of the health risks of SLT use.

*“As they (tobacco users) watch entertaining tik tok videos, they listen (to) songs, watch YouTube videos, if we will send such small videos it will be very good” (Counsellor, FGD)*

*“In my opinion everyone is using Facebook. You have to upload it in Facebook… you can use WhatsApp too” (Counsellor, FGD)*

*Visualized features:* Participants were in favour of image or pictorial mobile messages to advise against SLT, making it easy to convey the message to both literate and illiterate people.

*“Send photo…” (Male tobacco user, IDI)*

*“You send them pictures” (Counsellor, IDI)*

*“They do not have that much of patience to read whatever is written in the messages. So, if we have messages both pictorial and text based, that would be effective” (Physician, IDI)*

*Language of the messages*: Tobacco users opined that messages should be in the local language.

*“I don’t know how to read, it should be in Odia!” (Male tobacco user, IDI)*

*Length of the messages:* Physicians and counsellors insisted upon shorter and simple mobile messages.

*“Messages should be short and sweet” (Counsellor, FGD)*

*“It should be very short and simple. Simple language” (Physician, IDI)*

*Frequency and time of the messages*: Physicians and tobacco users mentioned that frequent messages can be annoying for users.

*“It shouldn’t be sent more frequently, otherwise they will get bored. They won’t see and read” (Physician, IDI)*

*“Message frequency should be once in 5 days or once in 6 days” (Male tobacco user, IDI)*

Tobacco users suggested that the messages should be shared in the evenings

*"4:30 to 5:30 in the evening is suitable because it is the post-lunch time at home" (Male tobacco user, IDI)*

## Discussion

The study findings reveal that the use of SLT is common in the community; however, the cessation efforts within the UPHC focused more on smoking. Peer factors accounted for both tobacco consumption as well as considering cessation. Tobacco users perceived the mobile phone counselling advice useful for quitting. Illiteracy and personal possession of mobile phones were identified as barriers to accessing mHealth interventions. Interestingly, the ease of messaging and the rising popularity of social media applications were identified as facilitators for mHealth interventions. Respondents strongly felt that messages should be tailored to individual's needs and be user-friendly. They also preferred pictorial, short and simple messages in the local language. We employed a formative research process involving representatives of the intended group of recipients of messages to inform the development of messages for SLT cessation.

Our findings are in line with previous research conducted by Murthy et al. in India that demonstrated that there is inadequate knowledge of the harm of SLT use among SLT users and HCPs. The above study also found a lack of awareness of SLT cessation interventions among both, users and providers.^21^ Our study provides information that SLT users are willing to use the mHealth counselling intervention. The users made significant recommendations for the mHealth intervention. They were interested in messages that took cognizance of health, family and financial perspectives. Such findings have also been reported by other studies that have evaluated smoking cessation interventions.^22,23^ A PCP suggested that messages should be context-specific and tailored according to the patients’ health condition, education and place of residence. Other studies have also reported the need to account for individual recipient differences while developing mHealth interventions for smoking cessation.^23,24^ Our findings suggest that mobile messages should be pictorial or a combination of text and picture. This concurs with other studies that used pictures or images through mobile messages to reach all users, including those unable to read.^25,26^ This may be instrumental in promoting cessation or reinforcing the decision to quit.

Counsellors felt that there was a potential for using social networks such as Facebook and WhatsApp for delivering messages for SLT cessation. Other studies have shown that smokers favoured smartphone-enabled social media applications such as WhatsApp and Snapchat for smoking cessation efforts.^22,27^ The suggestions of counsellors to incorporate information through social media is feasible. In India, there is an increasing availability and decreasing cost of the internet and smartphones.^28^

Our study recommends that the messages should reflect the harmful effect of SLT and the benefits of quitting SLT. This is not in accordance with a few other studies that have found participants’ preference for reframing the harm of smoking as a benefit of quitting.^22^ In a study by Luk et al., smokers reported that negative health warning messages may lead to feelings of defiance prompting them to smoke. They preferred messages describing the positive impact of quitting.^23^ Users in our study felt that the messages shouldn’t be very frequent (not more than once in 4 days). A study conducted on African-American patients with diabetes also showed a similar result where the majority of participants were in favour of receiving weekly text messages and they preferred short text messages.^29^ More frequent messages could be viewed as annoying and similar to ‘spam’ e-mails and could induce dropout from the program.^30^ Findings from a few formative research studies^25,31^ show that participants prefer a neutral and positive tone in the messages targeting behaviour change. These findings contradict our study finding that indicated that the users felt that the mobile messages should create feelings of anxiety to influence users to quit SLT. However, other studies in Low and Middle Income Countries (LMICs) show that graphic and strong emotional messages linked to the serious consequences of smoking are more likely to be effective with smokers.^32^ We feel there is an ambiguity in these recommendations and further research on the optimal use of fear in message-based SLT cessation programs is needed since the present findings suggest a degree of variability in the personal preferences of users and HCPs.

There is increasing evidence that text messaging programs can help people modify their health behaviours. The evidence is particularly strong for smoking cessation and has the potential to be adapted for SLT. A major strength of the study is the triangulation of data obtained from HCPs and SLT users along with the use of different methods of qualitative inquiry. Other methodological strengths are the inclusion of diverse tobacco users consuming different types of SLT products. There may, however, be a risk that the data was influenced by sampling bias as it was collected from a small number of participants from a few urban clinics. While we collected data on the quantity and frequency of SLT use we did not use it as an inclusion or sampling criterion which may affect the application of the intervention since needs of occasional users are likely to differ from regular or heavy users.

### Implications

Screening for tobacco use during routine health interactions and subsequent personalized advice or interventions offered is opportunistic, as the identified tobacco user might not be seeking cessation support. Tobacco cessation services delivered in primary care settings in India thus have the potential to reach a large number of tobacco users and reduce the cost of long-term care. Adding the component of well-designed messages through mHealth will be cost-effective and help improve the chance of quitting.

## Conclusion

The use of formative research helped in the collection of diverse perceptions of stakeholders. These findings will be used to devise and implement an intervention for the SLT user population who are availing of primary care services. Important themes revealed from the formative research will be used to inform a mHealth message-based SLT intervention.

## Supplemental Material

sj-docx-1-dhj-10.1177_20552076221150581 - Supplemental material for Designing a mobile health smokeless tobacco cessation intervention in Odisha, India: User and provider perspectives Click here for additional data file. Supplemental material, sj-docx-1-dhj-10.1177_20552076221150581 for Designing a mobile health smokeless tobacco cessation intervention in Odisha, India: User and provider perspectives by Rajmohan Panda, Supriya Lahoti, Arti Mishra, Rajath R Prabhu, Sangeeta Das, Durga Madhab Satapathy and Irwin Nazareth in Digital Health

sj-docx-2-dhj-10.1177_20552076221150581 - Supplemental material for Designing a mobile health smokeless tobacco cessation intervention in Odisha, India: User and provider perspectives Click here for additional data file. Supplemental material, sj-docx-2-dhj-10.1177_20552076221150581 for Designing a mobile health smokeless tobacco cessation intervention in Odisha, India: User and provider perspectives by Rajmohan Panda, Supriya Lahoti, Arti Mishra, Rajath R Prabhu, Sangeeta Das, Durga Madhab Satapathy and Irwin Nazareth in Digital Health

sj-docx-3-dhj-10.1177_20552076221150581 - Supplemental material for Designing a mobile health smokeless tobacco cessation intervention in Odisha, India: User and provider perspectives Click here for additional data file. Supplemental material, sj-docx-3-dhj-10.1177_20552076221150581 for Designing a mobile health smokeless tobacco cessation intervention in Odisha, India: User and provider perspectives by Rajmohan Panda, Supriya Lahoti, Arti Mishra, Rajath R Prabhu, Sangeeta Das, Durga Madhab Satapathy and Irwin Nazareth in Digital Health

sj-docx-4-dhj-10.1177_20552076221150581 - Supplemental material for Designing a mobile health smokeless tobacco cessation intervention in Odisha, India: User and provider perspectives Click here for additional data file. Supplemental material, sj-docx-4-dhj-10.1177_20552076221150581 for Designing a mobile health smokeless tobacco cessation intervention in Odisha, India: User and provider perspectives by Rajmohan Panda, Supriya Lahoti, Arti Mishra, Rajath R Prabhu, Sangeeta Das, Durga Madhab Satapathy and Irwin Nazareth in Digital Health

sj-docx-5-dhj-10.1177_20552076221150581 - Supplemental material for Designing a mobile health smokeless tobacco cessation intervention in Odisha, India: User and provider perspectives Click here for additional data file. Supplemental material, sj-docx-5-dhj-10.1177_20552076221150581 for Designing a mobile health smokeless tobacco cessation intervention in Odisha, India: User and provider perspectives by Rajmohan Panda, Supriya Lahoti, Arti Mishra, Rajath R Prabhu, Sangeeta Das, Durga Madhab Satapathy and Irwin Nazareth in Digital Health
