# Supplementary material for: Designing a mobile health smokeless tobacco cessation intervention in Odisha, India: User and provider perspectives
Source: Digit Health. 2023 Jan 11;9:20552076221150581. doi: 10.1177/20552076221150581 (PMC9841872; doi:10.1177/20552076221150581)
Supplement: sj-docx-1-dhj-10.1177_20552076221150581 - Supplemental material for Designing a mobile health smokeless tobacco cessation intervention in Odisha, India: User and provider perspectives [file sj-docx-1-dhj-10.1177_20552076221150581.docx]

**ANNEXURE 1. INTERVIEW GUIDE FOR TOBACCO USERS VISITING PRIMARY CARE**

**INSTRUCTION:**

**NOTE:** This tool has been developed for the in-depth interview of participant’s who were tobacco users visiting primary care clinics. Each section of the tool is to be filled carefully. The interview should only continue after reading out the entire information sheet to the participant while ensuring that information has been understood and the written consent from the participant has been secured. Completion of the interview using the tool should take between 25- 40 minutes (depending on the participant’s reply).

**Participant data:**

Age (in years):

Sex:

Highest level of education completed:

Marital status:

Religion:

Community:

Residence:

Monthly family income:

**Questions:**

1. Let’s start the discussion by talking about your habit of using tobacco?

**Probes:**

- Type of tobacco use
- Duration of tobacco use
- Reason for initiation and age of initiation

1. Have you considered quitting tobacco use? If so, why? What steps have you taken to quit tobacco? What factors contributed to your decision to quit and to your decision to continue tobacco?
2. Have you ever considered taking help from doctors/counselors to quit tobacco use? If yes, can you tell us more about the experience?
3. What do you think about a system where you can be helped to quit tobacco using mobile phone based counselling services? Can you elaborate your thoughts on its merits, demerits?

*In this research we have developed some messages which will be used in mobile phone based counselling services. These messages will be delivered using phone calls as well as texts. These have been developed talking suggestions from tobacco users of different age groups, sex, education, section of society, economic background and forms of tobacco. I will read out different messages that have been developed and then we will proceed further.*

“……………………………………………………………………………………………………………………………………………………………………………………………………………………………………………………………………………………………………………………………………………………………………”

5. So what are your first thoughts on these messages?

6. What do you think about the content of the messages? Did you understand the language and content of the messages?

1. Can you comment on the length of messages? How frequently should these messages be delivered through phone calls and texts?
2. Can you list the things that are not good about the messages?
3. What are the suggestions you would like to offer to make the messages more effective?

That concludes our interview. Thank you so much for coming and sharing your thoughts and opinions with us.
