# Supplementary material for: Designing a mobile health smokeless tobacco cessation intervention in Odisha, India: User and provider perspectives
Source: Digit Health. 2023 Jan 11;9:20552076221150581. doi: 10.1177/20552076221150581 (PMC9841872; doi:10.1177/20552076221150581)
Supplement: sj-docx-2-dhj-10.1177_20552076221150581 - Supplemental material for Designing a mobile health smokeless tobacco cessation intervention in Odisha, India: User and provider perspectives [file sj-docx-2-dhj-10.1177_20552076221150581.docx]

**ANNEXURE 2. INTERVIEW GUIDE FOR PHYSICIANS WORKING IN PRIMARY CARE**

NOTE: This tool has been developed for the in-depth interview of physicians who were working at the primary care clinics. Each section of the tool is to be filled carefully. The interview should only continue after reading out the entire information sheet to the participant while ensuring that information has been understood and the written consent from the participant has been secured. Completion of the interview using the tool should take between 25- 40 minutes (depending on the participant’s reply).

Please record the following information:

- Age
- Gender
- Cadre/Designation
- Educational qualification (specialization if any)
- Years at this facility,
- Total years of experience

**Introduction** (the section below should be read out by the facilitator and ensure that the participants understand the same)

Thank you for agreeing to participate. The reason for conducting these discussions is that we can get more in-depth information from you. We are very interested to hear your valuable opinion on the “scripts/messages” for calls as well as texts, developed for the mobile counselling services which will be used in helping tobacco users quit tobacco*.*

**Questions:**

1. Let’s start the discussion by talking tobacco use in the country, state, district and community?
2. What are your thoughts on the tobacco cessation methods? Are you aware of various methods of tobacco cessation? Can you elaborate on the type of cessation services you are aware of?
3. What is the type of cessation services available for the patients visiting your health facility?
4. What do you think about a system where tobacco users can be helped to quit tobacco using mobile phone-based counselling? Can you elaborate your thoughts on its merits, demerits?

*In this research we have developed some messages which will be used in mobile phone based counselling services. These messages will be delivered using phone calls as well as texts. These have been developed talking suggestions from tobacco users of different age groups, sex, education, section of society, economic background and forms of tobacco. I will read out different messages that have been developed and then we will proceed further.*

“……………………………………………………………………………………………………………………………………………………………………………………………………………………………………………………………………………………………………………………………………………………………………”

5. So what are your first thoughts on these messages to be used in phone calls and texts?

6. What do you think about the content of the messages? Did you understand the language and content of the messages?

1. Can you comment on the length of messages? How frequently should the tobacco users receive these messages through phone calls and texts?
2. Can you list the things that are not good about the messages?
3. What are the suggestions you would like to offer to make the messages more effective?

That concludes our interview. Thank you so much for coming and sharing your thoughts and opinions with us.
