# Supplementary material for: Designing a mobile health smokeless tobacco cessation intervention in Odisha, India: User and provider perspectives
Source: Digit Health. 2023 Jan 11;9:20552076221150581. doi: 10.1177/20552076221150581 (PMC9841872; doi:10.1177/20552076221150581)
Supplement: sj-docx-3-dhj-10.1177_20552076221150581 - Supplemental material for Designing a mobile health smokeless tobacco cessation intervention in Odisha, India: User and provider perspectives [file sj-docx-3-dhj-10.1177_20552076221150581.docx]

**ANNEXURE 3. FOCUS GROUP DISCUSSION GUIDE FOR COUNSELLORS WORKING IN PRIMARY CARE**

**INSTRUCTION:**

Consent forms for focus group participants should be completed in advance by all those seeking to participate. Below is a summary of the information facilitators should use to make sure participants understand the information in the consent form. Record the following information from each participant by a circulating a sign-in sheet with a few quick demographic questions.

- Age
- Gender
- Cadre/Designation
- Educational qualification (specialization if any)
- Years at this facility,
- Total years of experience

**Introduction** (the section below should be read out by the facilitator and ensure that the participants understand the same)

*Thank you for agreeing to participate. We are very interested to hear your valuable opinion on the “scripts/messages” for calls as well as texts, developed for the mobile counselling services which will be used in helping tobacco users quit tobacco.*

1. Explanation of the process: The discussion we are going to have today is called as focus group discussion. As you can see we have a gathering of diverse group of people with one common factor, i.e. all of you are health providers.

- Through this discussion we will learn from you positive and negative points on the developed messages for the mobile tobacco cessation services.
- You must remember that we are not trying to achieve consensus, we’re gathering information
- The reason for conducting these discussions is that we can get more in-depth information from a smaller group of people in focus groups. This allows us to understand the context behind the answers and helps us explore solutions in more detail.

***Please note***

- Focus group will last about one hour
- Feel free to move around

1. Ground Rules

- Everyone should participate.
- Information provided in the focus group must be kept confidential
- Stay with the group and please don’t have side conversations
- Turn off cell phones
- Have fun

*Do you have any questions?*

1. **Turn on the Recorder**
2. Introductions

*Discussion begins, make sure to give people time to think before answering the questions and don’t move too quickly. Use the probes to make sure that all issues are addressed, but move on when you feel you are starting to hear repetitive information.*

**Questions:**

1. Let’s start the discussion by talking tobacco use in the country, state, district and community?
2. What are your thoughts on the tobacco cessation methods? Are you aware of various methods of tobacco cessation? Can you elaborate on the type of cessation services you are aware of?
3. What are the type of cessation services available for the patients visiting your health facility?
4. What do you think about a system where tobacco users can be helped to quit tobacco using mobile phone based counselling? Can you elaborate your thoughts on its merits, demerits?

*In this research we have developed some messages which will be used in mobile phone based counselling services. These messages will be delivered using phone calls as well as texts. These have been developed talking suggestions from tobacco users of different age groups, sex, education, section of society, economic background and forms of tobacco. I will read out different messages that have been developed and then we will proceed further.*

“……………………………………………………………………………………………………………………………………………………………………………………………………………………………………………………………………………………………………………………………………………………………………”

5. So what are your first thoughts on these messages to be used in phone calls and texts?

6. What do you think about the content of the messages? Did you understand the language and content of the messages?

1. Can you comment on the length of messages? How frequently should the tobacco users receive these messages through phone calls and texts?
2. Can you list the things that are not good about the messages?
3. What are the suggestions you would like to offer to make the messages more effective?

That concludes our discussion group. Thank you so much for coming and sharing your thoughts and opinions with us.
