# Supplementary material for: Designing a mobile health smokeless tobacco cessation intervention in Odisha, India: User and provider perspectives
Source: Digit Health. 2023 Jan 11;9:20552076221150581. doi: 10.1177/20552076221150581 (PMC9841872; doi:10.1177/20552076221150581)
Supplement: sj-docx-4-dhj-10.1177_20552076221150581 - Supplemental material for Designing a mobile health smokeless tobacco cessation intervention in Odisha, India: User and provider perspectives [file sj-docx-4-dhj-10.1177_20552076221150581.docx]

**Appendix 4: Study participants and key areas covered**

| Study Participants | Data Collection | Age Range | Key Areas Covered |
| --- | --- | --- | --- |
| Primary care physicians at the UPHCs | 5 IDIs  (3 Female, 2 Male) | 40-60 yrs. | - Current understanding of the health risk of SLT use. - The perceived value of text messages and acceptability of messages. - The challenges faced by SLT users in quitting SLT. |
| Counsellors at UPHCs | 2 FGDs  (6 counsellors each group 8 female, 4 male) | 20-50 yrs. |  |
| Tobacco users visiting UPHCs | 26 IDIs  (19 males, 7 females) | 30-70 yrs. | - SLT use information from tobacco users. - Current understanding of the health risk of SLT use among SLT users - The value of text messages and acceptability of messages - Views about counselling against SLT use by health care providers. |
