# Supplementary material for: Designing a mobile health smokeless tobacco cessation intervention in Odisha, India: User and provider perspectives
Source: Digit Health. 2023 Jan 11;9:20552076221150581. doi: 10.1177/20552076221150581 (PMC9841872; doi:10.1177/20552076221150581)
Supplement: sj-docx-5-dhj-10.1177_20552076221150581 - Supplemental material for Designing a mobile health smokeless tobacco cessation intervention in Odisha, India: User and provider perspectives [file sj-docx-5-dhj-10.1177_20552076221150581.docx]

**COREQ Checklist**

| Topic | Item no. | Guide Questions/Description | Response/ Reported on pg. no |
| --- | --- | --- | --- |
| **Domain 1: Research team and reﬂexivity** | | | |
| *Personal characteristics* | | | |
| Interviewer/facilitator | 1 | Which author/s conducted the interview or focus group? | 4 |
| Credentials | 2 | What were the researcher’s credentials? E.g. PhD, MD | 4 |
| Occupation | 3 | What was their occupation at the time of the study? | 4 |
| Gender | 4 | Was the researcher male or female? | NA  Not relevant to this study as it would not have affected the conduct of the In-depth interviews and Focus Group Discussions. Both male and female researchers were involved |
| Experience and training | 5 | What experience or training did the researcher have? | 4 |
| *Relationship with participants* | | | |
| Relationship established | 6 | Was a relationship established prior to study commencement? | NA  Since the relationship between the participant and investigator was professional |
| Participant knowledge of the interviewer | 7 | What did the participants know about the researcher? e.g. personal goals, reasons for doing the research | NA  Details such as rationale of the study, the research group’s work and about the researchers involved in the study were provided in the Participant Information Sheet (PIS) |
| Interviewer characteristics | 8 | What characteristics were reported about the interviewer/facilitator?  e.g. Bias, assumptions, reasons and interests in the research topic | 4  The interest of the investigators has been reported |
| **Domain 2: Study design** | | | |
| *Theoretical framework* | | | |
| Methodological orientation and Theory | 9 | What methodological orientation was stated to underpin the study? e.g. grounded theory, discourse analysis, ethnography, phenomenology,  content analysis | 3 (Study design)  4,5 (Data analysis) |
| *Participant selection* |  |  |  |
| Sampling | 10 | How were participants selected? e.g. purposive, convenience, consecutive, snowball | 3,4 |
| Method of approach | 11 | How were participants approached? e.g. face-to-face, telephone, mail,  email | 4 |
| Sample size | 12 | How many participants were in the study? | 3 |
| Non-participation | 13 | How many people refused to participate or dropped out? Reasons? | 5 |
| *Setting* |  |  |  |
| Setting of data collection | 14 | Where was the data collected? e.g. home, clinic, workplace | 4 |
| Presence of non- participants | 15 | Was anyone else present besides the participants and researchers? | NA- no other person was present |
| Description of sample | 16 | What are the important characteristics of the sample? e.g. demographic  data, date | 5 |
| *Data collection* | | | |
| Interview guide | 17 | Were questions, prompts, guides provided by the authors? Was it pilot tested? | 4  Topic guides included in Annexure 1, 2, and 3. |
| Repeat interviews | 18 | Were repeat inter views carried out? If yes, how many? | NA  No repeat interviews were carried out |
| Audio/visual recording | 19 | Did the research use audio or visual recording to collect the data? | 4 |
| Field notes | 20 | Were field notes made during and/or after the interview or focus group? | 4 |
| Duration | 21 | What was the duration of the inter views or focus group? | 4 |
| Data saturation | 22 | Was data saturation discussed? | 4,5 |
| Transcripts returned | 23 | Were transcripts returned to participants for comment and/or correction? | NA  The transcripts were not returned to participants |
| **Domain 3: analysis and ﬁndings** | | | |
| *Data analysis* | | | |
| Number of data coders | 24 | How many data coders coded the data? | 5 |
| Description of the coding  tree | 25 | Did authors provide a description of the coding tree? | NA |
| Derivation of themes | 26 | Were themes identified in advance or derived from the data? | 5 |
| Software | 27 | What software, if applicable, was used to manage the data? | 5 |
| Participant checking | 28 | Did participants provide feedback on the findings? | NA  The participants did not provide feedback on the findings |
| *Reporting* | | | |
| Quotations presented | 29 | Were participant quotations presented to illustrate the themes/findings?  Was each quotation identified? e.g. participant number | Pages 6-12 |
| Data and findings consistent | 30 | Was there consistency between the data presented and the findings? | Page 6-12  Quotations are presented alongside their interpretations to ensure consistency |
| Clarity of major themes | 31 | Were major themes clearly presented in the findings? | 5,6 (Table 1)  Pages 6-12 (Results) |
| Clarity of minor themes | 32 | Is there a description of diverse cases or discussion of minor themes? | 5,6 (Table 1)  Pages 6-12 (Results) |
